# Prevalence of Coccidiosis and Its Associated Risk Factors Among Calves in and Around Haramaya Town, Eastern Hararge, Oromia, Ethiopia

**DOI:** 10.1002/vms3.71245

**Published:** 2026-09-24

**Authors:** Balcha Tadesse Jiru, Ayechew Yetayeh Emiru

**Affiliations:** ^1^ School of Veterinary Medicine Woldia University Woldia Ethiopia

**Keywords:** calves, coccidiosis, Eimeria, Haramaya, prevalence

## Abstract

**Background:**

Coccidiosis is a protozoan disease caused by single‐celled parasites of the genus *Eimeria*. It occurs worldwide and is a common cause of enteric disease in calves, resulting in economic losses due to reduced productivity and increased management costs. This study was conducted to determine the prevalence of calf coccidiosis and assess associated risk factors in and around Haramaya, East Hararghe Zone, Ethiopia.

**Methods:**

A cross‐sectional study was conducted on 284 calves in and around Haramaya. Faecal samples were collected and examined for *Eimeria* oocysts using a centrifugal faecal flotation technique with saturated sugar solution. Potential risk factors, including age, sex, breed, body condition score, farm hygiene status, management practices and study kebeles, were assessed. Data were analysed to determine associations between infection status and the hypothesised risk factors.

**Results:**

Of the 284 calves examined, 64 were positive for *Eimeria* oocysts, yielding an overall prevalence of 22.5% (95% CI: 18.1–27.8). Sex, breed, study kebeles and management practices were not significantly associated with the prevalence of coccidiosis (*P* > 0.05). However, farm hygiene status showed a significant association (*P* < 0.05), with a prevalence of 31.3% in farms with poor hygiene compared to 14.6% in farms with good hygiene. Body condition score was also significantly associated with infection (*P* < 0.05), with higher prevalence observed in calves with poor body condition. In addition, calves aged 6 to <12 months showed a significantly higher prevalence than calves younger than six months (*P* < 0.05).

**Conclusions:**

Coccidiosis is a prevalent disease of calves in and around Haramaya. Older calves, poor body condition and inadequate farm hygiene were identified as important risk factors associated with infection. Improving farm hygiene and implementing appropriate prevention and treatment strategies are recommended to reduce the burden of coccidiosis in calves.

## Graphical abstract

Calf coccidiosis caused by Eimeria spp. had a prevalence of 22.5% (95% CI: 18.1–27.8) in Haramaya, Ethiopia. The prevalence of coccidiosis was higher among calves aged 6 months to <12 months (27.3%) than those aged <6 months (17.2%), among calves with poor body condition (29.7%) than those with good body condition (18.6%), and on farms with poor hygiene (31.3%). Infection was significantly associated with poor farm hygiene, poor body condition and age 6 months to < 12 months (*P* < 0.05), while sex, breed, origin and management practices showed no association. Strengthening hygiene measures and implementing strategic control programmes are critical to reducing disease burden in calves.

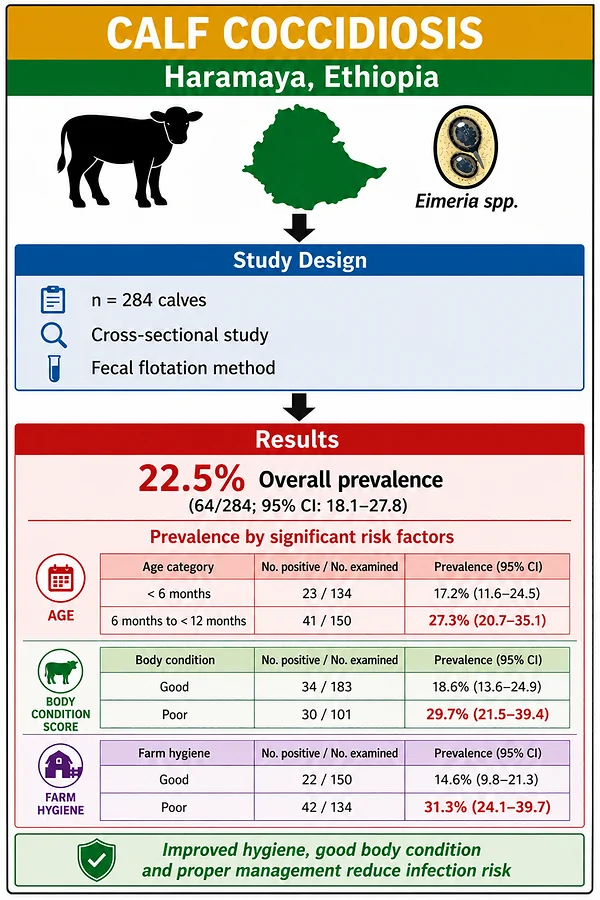

## 1 Introduction

Ethiopia possesses the largest livestock population in Africa. According to the Central Statistics Agency (CSA 2021) livestock sample survey, the national herd comprises approximately 70.3 million cattle, 42.9 million sheep, 52.5 million goats and 8.1 million camels. Livestock production plays a pivotal role in the national economy by supplying animal‐source protein, draft power for crop cultivation, transportation services, export commodities, organic fertiliser and household energy, while also serving as a financial buffer during periods of crop failure and as a means of capital accumulation (CSA 2020). Economically, the sector accounts for up to 40% of agricultural gross domestic product (GDP), nearly 20% of total GDP and approximately 20% of national foreign exchange earnings (World Bank 2017).

Livestock productivity in Ethiopia is constrained by numerous infectious and parasitic diseases, among which coccidiosis is particularly significant. Coccidiosis is caused by one or more species of coccidia and represents a serious enteric disorder affecting a wide range of domestic animals. The disease is characterised by acute invasion and destruction of the intestinal mucosa, leading to diarrhoea, weight loss, fever, anorexia, emaciation and, in severe cases, mortality (Mogos and Walkite 2018). It is a protozoan parasitic disease resulting from infection with intracellular organisms that colonise the epithelial cells of the intestinal tract and is recognised as one of the most prevalent and economically important diseases of cattle worldwide (Pence 2011).

Bovine coccidiosis is of considerable importance to cattle health and productivity. Although animals of all age groups are susceptible, clinical eimeriosis occurs predominantly in young stock. In many cases, infection remains subclinical, yet it contributes to substantial economic losses through reduced feed intake, poor weight gain, impaired feed conversion efficiency, unthriftiness, diarrhoea, dysentery, anaemia and increased predisposition to secondary infections (Bahrami and Alborzi 2013).

More than twelve *Eimeria* species have been identified in cattle and buffalo to date, with *Eimeria* bovis, *Eimeria zuernii* and *Eimeria auburnensis* most frequently reported (Lassen et al. 2009). Among these, *E. bovis* and *E. zuernii* are highly pathogenic and are responsible for significant morbidity and mortality due to severe disruption of intestinal absorptive functions. *Eimeria* species exhibit marked host specificity, which restricts cross‐species transmission and confines infection largely to cattle, with only occasional passive mechanical dissemination of oocysts (Alemayehu et al. 2013).

Calves acquire infection primarily through ingestion of sporulated oocysts, and transmission is facilitated under conditions of communal housing and overcrowding. Additionally, contaminated udders may serve as a source of infection from cow to calf (Faber et al. 2002). Clinically affected animals may exhibit profuse, watery to haemorrhagic diarrhoea lasting approximately one week or softer faeces containing mucus and sloughed intestinal epithelium; in severe cases, progressive anaemia may develop (Coetzer and Justin 2004).

*Eimeria* oocysts require an external sporulation period, ranging from several days to weeks depending on species, temperature, humidity and other environmental conditions. These oocysts exhibit remarkable environmental resistance and can survive prolonged freezing, tolerating temperatures as low as −18°C for extended periods, including throughout the winter season (Daugschies and Najdrowski 2005).

The manifestation of clinical coccidiosis in cattle is influenced by multiple determinants, including the infecting *Eimeria* species, host age, infective dose (number of ingested oocysts), concurrent infections and prevailing production and management systems. Although clinical cases are more apparent, subclinical coccidiosis is economically more consequential, accounting for over 95% of total production losses associated with the disease. Annual economic losses have been estimated to exceed US$400 million globally, and growth retardation of up to two months in affected calves has been reported (Mogos and Walkite 2018).

The epidemiology and species distribution of bovine coccidiosis have been documented in several countries; however, data on morbidity and mortality remain limited in Ethiopia. In Eastern Hararghe, only a few investigations have been conducted. Reported prevalence rates include 22.7% in dairy farms in Dire Dawa (Mehreteab et al. 2012), 24.4% in Haramaya (Walkite et al. 2018) and 31.8% in Haramaya district (Mohamed et al. 2017). Consequently, there remains a paucity of comprehensive information regarding the occurrence, associated risk factors and production losses attributable to bovine coccidiosis in Eastern Hararghe Zone. Moreover, no detailed investigation has specifically addressed the prevalence and associated risk factors of coccidiosis in calves. Therefore, the present study was designed to determine the prevalence of calf coccidiosis and to identify its associated risk factors in and around Haramaya town, Eastern Hararghe, Oromia, Ethiopia.

## 2 Materials and Methods

### 2.1 Study Area

The study was conducted in Haramaya town and five surrounding kebeles (Tuji Gabisa, Ifa Haramaya, Biftu Gada, Bate and Adele) in Haramaya District, East Hararghe Zone, Oromia Regional State, eastern Ethiopia. Haramaya District is located approximately 500 km east of Addis Ababa and about 18 km west of Harar, between 1600 and 2100 m above sea level. The district lies approximately between 9°09′ and 9°32′ N latitude and 41°50′ and 42°05′ E longitude. It receives a bimodal rainfall pattern with annual rainfall ranging from approximately 492 to 866 mm and annual temperatures ranging from 5.2°C to 24°C. Mixed crop–livestock farming is the dominant production system, with cattle production serving as one of the principal agricultural enterprises. Haramaya District consists of 33 rural kebeles and three urban kebeles, and livestock production is an integral component of household livelihoods. (Gilo and Berta 2016).

**FIGURE 1:**
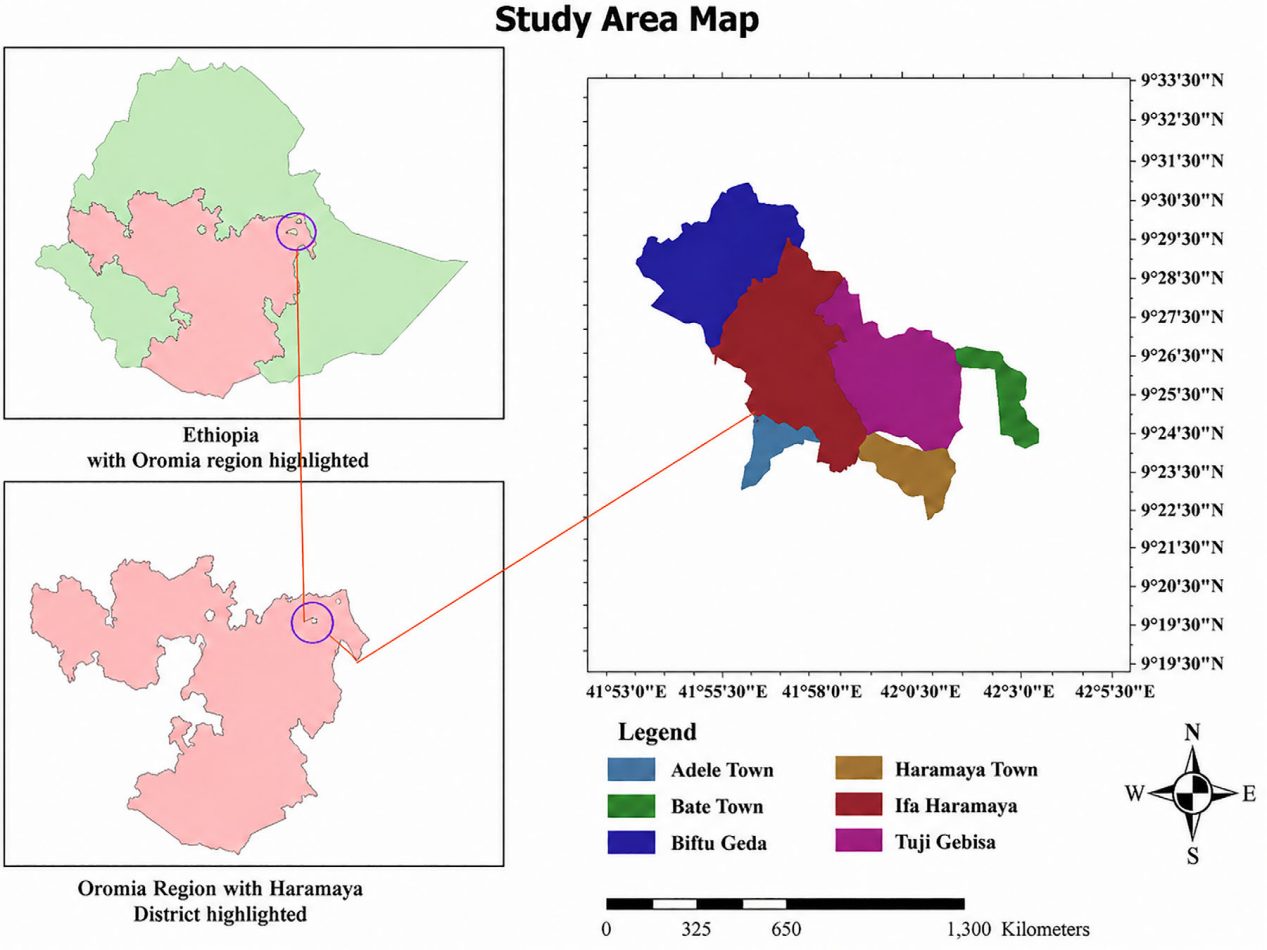
Map showing the location of Haramaya District in East Hararghe Zone, Oromia Regional State, eastern Ethiopia and the study sites in Haramaya town and the five surrounding kebeles: Tuji Gabisa, Ifa Haramaya, Biftu Gada, Bate and Adele.

Haramaya town was purposively selected as the major urban area, while the surrounding Kebeles represented peri‐urban dairy production systems in the district. The surrounding kebeles (Tuji Gabisa, Ifa haramaya, Biftu Gada, Bate and Adele) were selected by simple random sampling from the list of kebeles surrounding Haramaya town (Figure 1). These kebeles were considered representative of the prevailing rural calf production and management systems in the study area. In this study, the term ‘Kebele’ refers to a local administrative unit within the Ethiopian administrative structure.

### 2.2 Study Animals

The study animals comprised crossbred (Holstein Friesian × indigenous cattle) and local breed calves less than 12 months of age. Based on age, the calves were categorised into two groups: younger calves (<6 months) and older calves (6 to <12 months). The age of each calf was determined based on information provided by the owner. Epidemiological information was collected with respect to their age, sex, breed, management system, farm hygiene, body condition, date of sample collection and kebele or name of the farm.

### 2.3 Study Design

A cross‐sectional study was conducted in and around Haramaya town, East Hararghe Zone, Oromia, Ethiopia. The overall study was carried out from November 2024 to May 2025, encompassing study preparation, farm registration, development of the sampling frame, field sample collection, laboratory examination, data management and statistical analysis. The cross‐sectional field sampling was conducted during the dry season to determine the prevalence of calf coccidiosis and its associated risk factors.

### 2.4 Sample Size Determination

The animals were selected by using simple random sampling method. Sample size was determined by setting 24.4% expected prevalence taken from the previous research worked by (Walkite et al. 2018). The desired sample size for the study was calculated using the formula given by (Thrusfield 2007).

$$N=\frac{1.96^{2}\;\mathrm{Pexp}\left(1-\mathrm{Pexp}\right)}{d^{2}}$$

where N = required sample size, Pexp = expected prevalence and d = desired absolute precision. The sample size for this work was determined using 1.96^2^ = z‐ value for a 95% confidence interval. Therefore, a total of 284 calves were sampled to determine the prevalence of calf coccidiosis in the study area.

### 2.5 Assessment of Management System and Farm Hygiene

Management system and farm hygiene status were assessed through a combination of a structured questionnaire and direct on‐farm observation. Farms were classified as intensive or extensive based on housing, feeding, grazing and overall husbandry practices following the general descriptions of Radostits et al. (2007). Intensive farms were characterised by permanent housing, stall feeding, controlled feeding and watering and regular sanitation practices. Extensive farms relied predominantly on free grazing with minimal housing and limited supplementary feeding. The study included intensive and extensive production systems, as no semi‐intensive farms were identified in the study area during the study period.

Farm hygiene status was evaluated by direct observation of calf housing and the surrounding environment using predefined criteria adapted from previous studies (Belay and Mekibib 2022). The assessment included the cleanliness of calf pens, accumulation of faeces, bedding condition, drainage, frequency of manure removal and the cleanliness of feed and water troughs. Farms were classified as having good hygiene when calf pens were clean and dry, manure was removed regularly, bedding was clean, drainage was adequate and feeding and watering facilities were well maintained. Poor hygiene was assigned when excessive faecal contamination, wet or dirty bedding, poor drainage, infrequent manure removal and visibly unhygienic feeding or watering facilities were observed.

### 2.6 Study Methodology

#### 2.6.1 Sampling Procedure

A community‐based cross‐sectional study was conducted to determine the prevalence of calf coccidiosis and associated risk factors. A multistage sampling procedure was employed.

In the first stage, Haramaya town was purposively selected because of its importance as the principal urban and peri‐urban dairy production area within the district. Subsequently, five surrounding kebeles (Tuji Gabisa, Ifa Oromia, Biftu Gada, Bate and Adele) were selected by simple random sampling from the list of kebeles surrounding Haramaya town.

In the second stage, a preliminary census was conducted in each selected study site. All farms keeping calves younger than 12 months of age were visited and registered with the assistance of local livestock development agents. During this census, all eligible calves were enumerated, and the resulting list of calves constituted the sampling frame for the study.

In the third stage, individual calves were selected from the sampling frame using a simple random sampling technique (lottery draw). Consequently, every eligible calf within the selected study sites had an equal probability of being included in the study. This approach minimised selection bias and ensured representative sampling of calves from both urban and peri‐urban production systems.

A total of 284 calves were examined, comprising 65 (22.9%) from Haramaya town, 47 (16.5%) from Tuji Gabisa, 39 (13.7%) from Ifa Oromia, 43 (15.1%) from Biftu Gada, 44 (15.5%) from Bate and 46 (16.2%) from Adele.

#### 2.6.2 Sample Collection and Transportation

All faecal samples and animal‐ and farm‐level data were collected by the authors using a standardised data collection sheet. Both authors are qualified veterinarians, and the same investigators performed all field sampling and data recording to ensure consistency in data collection.

Immediately after collection, fresh faecal samples were placed in clean, labelled, leak‐proof plastic containers and transported to the laboratory in an insulated cool box containing ice packs (approximately 4°C–8°C). Samples were processed using the flotation technique on the day of collection. When immediate processing was not possible, they were refrigerated at 4°C and examined within 24 h to preserve the morphology of the coccidian oocysts.

For laboratory examination, 3 g of each faecal sample was weighed using an analytical balance and transferred into a mortar. The sample was homogenised with a pestle, after which 40 mL of sugar flotation solution (specific gravity 1.27) was added and thoroughly mixed. The suspension was filtered through a strainer into a 100‐mL glass beaker and subsequently transferred into 15‐mL centrifuge tubes without filling to capacity. Following centrifugation at 1500 rpm for 5 min, additional flotation solution was added to form a convex meniscus at the rim of each tube. A clean coverslip was carefully placed on top and allowed to stand for 30 min. The coverslip was then gently lifted and mounted onto a clean glass slide, avoiding air bubble formation. The entire area under the coverslip was examined microscopically using a binocular microscope at 40× magnification (Dryden et al. 2005).

### 2.7 Data Management and Analysis

Data collected from study sites were entered and stored in a Microsoft excel spread sheet programme and coded for analysis. Statistical analysis was done on STATA version 20 statistical software. Categorical data were analysed first with the chi square (x^2^) test for independence as a screening process. A *P*‐value < 0.05 was considered as statistically significant.

## 3 Results

The present study revealed that, from a total of 284 calf faecal samples examined in the study area, 64 (22.5%) were positive for *Eimeria* spp. oocysts, with a 95% confidence interval (CI) of 18.1%–27.8%. Analysis of host‐related risk factors demonstrated significant associations (*P* < 0.05) between the occurrence of coccidiosis and age, body condition and farm hygiene. Although coccidian oocysts were detected across all age groups sampled, the highest prevalence was observed in calves aged 6 to < 12 months (27.3%) (X^2^ = 4.192, *p* = 0.041). Similarly, calves with poor body condition exhibited a higher prevalence (29.7%) compared to those with good body condition. The infection rate was also greater (31.3%) among calves raised under poor farm hygiene conditions (X^2^ = 4.613, *p* = 0.032) (Table 1).

**TABLE 1:** Prevalence of coccidiosis in calves with host related associated risk factors.

Risk factors	No. examined	No. positive (%)	CI 95%	X^2^	*P*‐value
**Sex**				2.27	0.131
Male	154	40 (26%)	19.6–33.5		
Female	130	24 (18.5%)	12.6–26.1		
**Total**	284	64 (22.5%)	18.1–27.8		
**Breeds**				0.793	0.373
Local	216	46 (21.3%)	16.3–27.3		
Cross	68	18 (26.5%)	17.3–38.3		
**Total**	284	64 (22.5%)	18.1–27.8		
**Age**				4.192	0.041
Less than 6 months	134	23 (17.2%)	11.6–24.5		
6 to < 12 months	150	41 (27.3%)	20.7–35.1		
**Total**	284	64 (22.5%)	18.1–27.8		
**Bodycondition**				4.613	0.032
Good	183	34 (18.6%)	13.6–24.9		
Poor	101	30 (29.7%)	21.5–39.4		
**Total**	284	64 (22.5%)	18.1–27.8		

In contrast, statistical analysis showed no significant association (*P* > 0.05) between coccidiosis prevalence and variables such as sex (X^2^ = 2.27, *p* = 0.131), breed (X^2^ = 0.793, *p* = 0.373), management system (X^2^ = 0.466, *p* = 0.495) and study kebeles of calves ([X^2^ = 1.316, *p* = 0.859], [as presented in Tables 1 and 2]).

**TABLE 2:** Prevalence of coccidian infections in calves studied in relation to farm practices, study kebeles and other management practices.

Risk factors	No. examined	No. positive (%)	CI 95%	X^2^	*P*‐value
**Farm hygiene**				11.275	0.001
Good	150	22 (14.6%)	9.8–21.3		
Poor	134	42 (31.3%)	24.1–39.7		
**Total**	284	64 (22.5%)	18.1–27.8		
**Management practices**				0.466	0.495
Extensive	167	40 (23.95%)	15.9–28.5		
Intensive	117	24 (20.5%)	17.1–32.6		
**Total**	284	64 (22.5%)	18.1–27.8		
**Study kebeles**				1.316	0.859
Haramaya town	65	23 (35.4%)	17.3 – 39.7		
Tuji gabisa	47	10 (21.2%)	15.1–37.5		
Ifa Haramaya	39	7 (17.9%)	14.2–41.7		
Biftu gada	43	7 (16.3%)	8.9–29.8		
Bate	44	7 (15.9%)	14.6–35.5		
Adele	46	10 (21.7%)	14.2–33.8		
**Total**	284	64 (22.5%)	18.1–27.8		

Coccidian infection in calves showed no statistically significant association with study kebeles or management practices, with chi‐square values of X^2^ = 1.316 (*p* = 0.859) and X^2^ = 0.466 (*p* = 0.495), respectively. However, a statistically significant difference was observed in the prevalence of coccidiosis in relation to farm hygiene (X^2^ = 11.275, *p* = 0.001) (Table 2).

## 4 Discussion

In the present study, coccidial oocysts were detected in 22.5% (64/284) of calves, demonstrating the occurrence of coccidial infection among calves in and around Haramaya town. The observed prevalence was comparable with reports from Dire Dawa (22.7%) (Mehreteab et al. 2012), Haramaya (24.4%) (Walkite et al. 2018), Wolaita Sodo (22.9%) (Zekarias and Shirge 2019), South Wollo (24.3%) (Temesgen 2016), Bahir Dar (24.9%) (Kassa et al. 1987) and Egypt (24.2%) (Nagwa et al. 2011). The similarity among these findings may reflect comparable calf management conditions, production systems, environmental contamination and other factors influencing the survival and transmission of Eimeria oocysts. In particular, inadequate sanitation, accumulation of faecal material and continuous exposure of calves to contaminated environments may contribute to maintaining coccidial transmission.

The prevalence observed in the present study was lower than reports from Sekachekorsa District, Jimma Zone (29.7%) (Zakir et al. 2023), Asella town (62.5%) (Asfaw et al. 2016), Addis Ababa and Debre Zeit (68.1%) (Abebe et al. 2008) and Kombolcha District of South Wollo (31.9%) (Alemayehu et al. 2013). Conversely, it was higher than reports from Debre Zeit (20%) (Kebadu 1998), Bahir Dar (20.1%) (Tamrat et al. 2020), Kacha Bira District (17.8%) (Mathewos and Endale 2024) and the Gondar area (21.1%) (Haile et al. 2019). Differences among studies may be related to variation in agroecological conditions, rainfall patterns, calf age distribution, farm management, hygiene status, stocking density, sampling strategies and diagnostic methods.

Previous evidence from the Haramaya area indicates that several Eimeria species circulate among calves. Solomon et al. (2017), who investigated calf coccidiosis in and around Haramaya District, identified six species, namely Eimeria bovis, E. zuernii, E. auburnensis, E. canadensis, E. ellipsoidalis and E. cylindrica. Among these, E. bovis (22.7%) and E. zuernii (17.2%) were the most frequently detected species, and mixed‐species infections were common. Similarly, in Kombolcha District of South Wollo, Alemayehu et al. (2013) identified E. bovis, E. zuernii, E. auburnensis, E. ellipsoidalis and E. alabamensis, with E. bovis and E. zuernii predominating. These findings provide useful regional context for the present study; however, species‐level comparison cannot be made because the current study was limited to detection of coccidial oocysts and did not identify Eimeria species. Therefore, the prevalence reported here should be interpreted as the proportion of calves positive for detectable coccidial oocysts rather than species‐specific Eimeria infection.

Sex was not associated with coccidial infection (χ^2^ = 2.27, p = 0.131), which agrees with findings reported by Walkite et al. (2018). The absence of an association suggests that male and female calves in the study area may have experienced similar exposure opportunities and management conditions. Similarly, breed was not associated with coccidial infection (χ^2^ = 0.793, *p* = 0.373), consistent with findings reported by Abebe et al. (2008) and Alemayehu et al. (2013). This may indicate that environmental exposure and management‐related factors have greater influence on infection occurrence than breed under the conditions of the present study. However, Zakir et al. (2023) reported an association between breed and infection, suggesting that the effect of breed may vary among study populations and management conditions.

Farm hygiene status was associated with coccidial infection (χ^2^ = 11.275, *p* = 0.001), with calves from farms classified as having poor hygiene showing a higher prevalence than those from farms with good hygiene. This finding agrees with reports by Mehreteab et al. (2012) and Asfaw et al. (2016). Poor hygiene may increase environmental contamination with Eimeria oocysts through accumulation of faeces in calf pens, contaminated bedding, inadequate cleaning and poor manure disposal. Overcrowding, poor ventilation and mixing calves of different ages may further increase opportunities for oocyst ingestion and transmission (Radostits et al. 2007). These findings emphasise the importance of appropriate calf housing sanitation and manure management in reducing exposure to coccidial oocysts.

Body condition was associated with coccidial infection (χ^2^ = 4.613, *p *= 0.032), with a higher prevalence observed among calves with poor body condition than among those with good body condition. Similar findings were reported by Mehreteab et al. (2012) and Asfaw et al. (2016), whereas Abebe et al. (2008) and Alemayehu et al. (2013) found no association. Poor nutritional status may impair immune competence and increase susceptibility to infection. However, because the present study was cross‐sectional, the direction of this association cannot be established. Poor body condition may increase susceptibility to coccidial infection, while coccidial infection may also contribute to impaired growth and body condition.

Age was associated with coccidial infection (χ^2^ = 4.192, *p* = 0.041), with a higher prevalence among calves aged 6 to <12 months than among calves younger than 6 months. Similar age‐related differences have been reported by Abebe et al. (2008), Mehreteab et al. (2012), Alemayehu et al. (2013) and Radostits et al. (2007). The higher prevalence among older calves may be related to their longer cumulative exposure to contaminated environments and greater contact with contaminated feed, water, bedding and other animals. However, age‐related differences may also reflect variations in management, nutrition and exposure patterns. Walkite et al. (2018) reported a different age pattern in calves from Haramaya University, Haramaya and Harar towns, suggesting that the relationship between age and coccidial infection may vary according to management and exposure conditions.

The management system was not associated with coccidial infection (χ^2^ = 0.466, p = 0.495), consistent with findings reported by Abebe et al. (2008), Alemayehu et al. (2013) and Temesgen (2016). The lack of association may reflect similarities in environmental exposure and hygiene conditions across the production systems included in the present study. However, this finding differs from Solomon et al. (2017), who reported an association between management system and coccidial infection in calves in and around Haramaya District. Such differences may be related to variation in farm structure, hygiene practices, stocking density, nutritional management and other local conditions.

An important limitation of the present study is that the detected coccidial oocysts were not identified to species level. Consequently, it was not possible to determine which Eimeria species were circulating in the study population or to distinguish potentially pathogenic species from non‐pathogenic or less pathogenic species. Although previous investigations in the Haramaya area have demonstrated the presence of several Eimeria species, including E. bovis and E. zuernii (Solomon et al. 2017), these species cannot be assumed to be responsible for the oocysts detected in the present study. Future studies incorporating sporulation‐based morphological identification and/or molecular characterisation, together with quantitative oocyst counts, would provide more detailed information on species composition, infection intensity and the potential clinical significance of coccidiosis among calves in the area.

## 5 Conclusion

The present study revealed that coccidial infection occurs among calves in and around Haramaya town, East Hararghe Zone, Ethiopia, with an overall prevalence of 22.5%. No statistically significant association was observed between coccidial infection and sex, breed, management system, or study kebele. However, age, body condition and farm hygiene status were significantly associated with infection occurrence. Calves aged 6 to <12 months, those with poor body condition, and calves from farms with poor hygiene conditions had a higher likelihood of infection. The findings indicate that farm‐level management factors, particularly hygiene practices, may play an important role in reducing exposure of calves to Eimeria oocysts. Therefore, improving calf housing sanitation, maintaining proper hygiene and strengthening routine health monitoring could contribute to reducing the occurrence of coccidial infection in calves. Further studies involving longitudinal follow‐up and broader geographical coverage are recommended to better understand infection dynamics and associated production impacts.

## Author Contributions

Both authors comprehended the study, designed and conducted all study procedures; analysed and interpreted experimental results and manuscript preparation. Both authors read and approved the final manuscript. AYE coordinated the responses to reviewers and revised the manuscript based on the reviewers’ comments.

## Funding

The authors have nothing to report.

## Ethics Statement

The study was conducted in accordance with accepted animal welfare principles. Faecal samples were collected directly from the rectum of calves by the researchers using standard veterinary procedures. Calves were handled humanely and minimally restrained only for the time required to obtain the faecal samples, with efforts made to minimise stress and discomfort. No experimental treatments, invasive procedures, or harmful interventions were performed. According to the applicable procedure of the College, research proposals involving experimental procedures with laboratory animals are submitted to the College Ethics Committee for ethical review and approval; the present field‐based study did not involve such experimental procedures. Before data collection, the objectives of the study were explained to calf owners or farm managers, and verbal informed consent was obtained before sample collection and questionnaire administration. Participation was voluntary, and information obtained from respondents was kept confidential.

## Conflicts of Interest

The authors declare no conflicts of interest.

## Data Availability

Data will be made available on request.
